# Reinventing gene expression connectivity through regulatory and spatial structural empowerment via principal node aggregation graph neural network

**DOI:** 10.1093/nar/gkae514

**Published:** 2024-06-17

**Authors:** Fengyao Yan, Limin Jiang, Danqian Chen, Michele Ceccarelli, Yan Guo

**Affiliations:** Department of Public Health and Sciences, University of Miami, Miami, FL 33126, USA; Department of Computer Science, University of South Carolina, Columbia, SC 29201, USA; Department of Public Health and Sciences, University of Miami, Miami, FL 33126, USA; Department of Public Health and Sciences, University of Miami, Miami, FL 33126, USA; Department of Public Health and Sciences, University of Miami, Miami, FL 33126, USA; Department of Public Health and Sciences, University of Miami, Miami, FL 33126, USA

## Abstract

The intricacies of the human genome, manifested as a complex network of genes, transcend conventional representations in text or numerical matrices. The intricate gene-to-gene relationships inherent in this complexity find a more suitable depiction in graph structures. In the pursuit of predicting gene expression, an endeavor shared by predecessors like the L1000 and Enformer methods, we introduce a novel spatial graph-neural network (GNN) approach. This innovative strategy incorporates graph features, encompassing both regulatory and structural elements. The regulatory elements include pair-wise gene correlation, biological pathways, protein–protein interaction networks, and transcription factor regulation. The spatial structural elements include chromosomal distance, histone modification and Hi-C inferred 3D genomic features. Principal Node Aggregation models, validated independently, emerge as frontrunners, demonstrating superior performance compared to traditional regression and other deep learning models. By embracing the spatial GNN paradigm, our method significantly advances the description of the intricate network of gene interactions, surpassing the performance, predictable scope, and initial requirements set by previous methods.

## Graphical Abstract

**Graphical Abstract:**
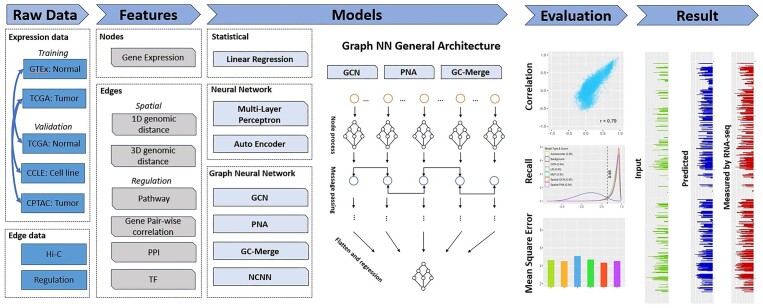

## Introduction

As one of the most scrutinized genomic features, gene expression functions as a critical link between an organism's genetic code and functional attributes. Its investigation is paramount in advancing our comprehension of biology, elucidating disease mechanisms, and fostering the development of targeted interventions for enhanced healthcare outcomes. It has been consistently observed that genes can manifest analogous expression patterns across diverse biological samples or conditions, forming an intrinsic web of connectivity. This observed phenomenon is underpinned by numerous factors, including shared regulatory elements, coexistence within common biological pathways, and the presence of feedback and feedforward loops, among others. The exploration of gene expression connectivity yields invaluable insights into the intricate regulatory networks and functional interplay among genes. The meticulous analysis of these patterns unveils the underlying biological processes and significantly contributes to our understanding of normal development, disease etiology and cellular responses to diverse stimuli.

Considerable efforts have been devoted to the exploration and practical application of gene expression connectivity. Notably, co-expression analysis has emerged as a prominent research field, yielding a wealth of impactful studies (1–3). Additionally, a dedicated focus has been placed on the aggregation of gene expression connectivity into reusable resources, exemplified by initiatives such as GeneFriends (4), ccNET (5), PlantNexus (6) and others. Among these endeavors, the Connectivity Map (CMAP) stands out as a particularly noteworthy achievement. CMAP furnishes a comprehensive compilation of gene expression data intricately linked with biological responses to various perturbations, encompassing interventions such as the introduction of drugs, small molecules, or genetic modifications (7). The inception of CMAP dates back nearly two decades to the nascent stages of high-throughput genomic technology. In 2017, a significant expansion of CMAP occurred, driven by the analysis of expression profiles from 1 million samples utilizing the L1000 method (8). The L1000 method, an integral component of the Library of Integrated Network-Based Cellular Signatures (LINCS) project by the National Health Institute, represents a high-throughput gene expression profiling technique. Succinctly, the L1000 method employs linear regression techniques to impute gene expression values for 11 350 genes based on the expression patterns of 955 landmark genes. Another notable tool, Enformer [ (9], utilizes transformers to incorporate long-range interactions to predict gene expression exclusively from genomic sequences. However, its utility is contingent on the availability of personalized genomes featuring individualized DNA sequences for accurate predictions. Consequently, the efficacy of Enformer is intricately tied to the feasibility of obtaining comprehensive and personalized genomic sequences, potentially restricting its applicability in scenarios where acquiring such data proves challenging or impractical. There have been other attempts of using graph features in gene expression prediction. For example, Multi-Layer Perceptron (MLP) has been modified into Neighbour Connection Neural Network (NCNN) for gene expression prediction using protein–protein interaction (PPI) as graph feature [ (10]. The tool GC-MERGE uses Graph Convolution Network (GCN) and Hi-C as graph feature for predicting gene expression [ (11].

While conventional statistical analyses often assume linearity, the intrinsic complexity of biological systems necessitates acknowledgment that gene interactions may unfold in non-linear patterns. For instance, in scenarios characterized by threshold responses, a gene's expression may undergo significant changes only upon surpassing a specific concentration threshold, as observed in microRNA regulation (12). Another illustrative example involves feedback loops, where the negative regulation of a gene's own expression product can give rise to non-linear dynamics and oscillations in gene expression levels (13). Our hypothesis posits that employing a linearity-agnostic approach may enhance the accuracy of capturing comprehensive associations among genes.

Within this study, we introduce an innovative method for gene expression prediction. Beyond conventional gene expression features, our approach integrates multiple graph features—encompassing both regulatory and structural elements—into a GNN. By exploiting the connectivity and relationships among these diverse features, our method enhances the predictive modeling of gene expression. Despite the integration of various feature types into a comprehensive graph representation, the practical application of the model necessitates only a minor segment of the transcriptome, as the remaining graph features are inferred from prior data. Rigorous independent validations substantiate that our graph-based approach surpasses the performance of preceding methods.

## Materials and methods

### Datasets and models

Gene expression data of 6903 samples from four consortiums were downloaded including Genotype-Tissue Expression Consortium (GTEx) [ (14], The Cancer Genome Atlas (TCGA) [ (15], Cancer Cell Line Encyclopedia (CCLE) [ (16], Clinical Proteomic Tumor Analysis Consortium (CPTAC) [ (17]. To ensure comprehensive comparison, we selected six models including the traditional linear regression, and four neural network models ranging from conventional to advanced including Multi-Layer Perceptron (MLP), Auto Encoder (AE), Graph Convolution Network (GCN) (18), and Principal Node Aggregation (PNA) (19). Both GCN and PNA are GNN models. Overall, all the trained models are trained on the first dataset and independently tested on the second dataset.

### Non-graph and graph features

Five types of genomic features are utilized. These five features can be summarized into three categories: (i) a. conventional: gene expression; (ii) spatial: a. chromosomal genomic distance; b. histone modification; c. 3D genomic distance inferred from Hi-C; (iii) regulation: a. biological pathway; b. pair-wise gene correlation, c. protein–protein interaction (PPI) network; d. transcription factor (TF) regulation. Gene expressions can be applied to all models. Other features, due to their spatial and regulatory nature, are more suitable for GNN models. For pair-wise gene correlation, the edges are formed by creating Pearson correlation matrix for all genes in the dataset. The edges are then selected by filtering gene pairs with Pearson correlation coefficient >0.9. The pathway data for our project is acquired from the Comparative Toxicogenomics Database (CTD) project (20). Because the connection edges are not available in CTD, for any pathway with N genes, we added random 2N edges to connect genes. PPI networks were obtained from STRING (21). The 100 000 pairs of PPI with the highest combined score were selected. Transcription factor regulation information was obtained by combing data from DoRothEA [ (22]. A total of 223 642 TF regulation pairs with confidence scores A and B were selected.

The chromosomal genomic distances are defined as the number of nucleotides between the center bases of two genes, and are calculated using the following formula:


\begin{eqnarray*}D = |\frac{{\left( {{{m}_1} + \ {{n}_1}} \right)}}{2} - \ \frac{{\left( {{{m}_2} + \ {{n}_2}} \right)}}{2}\end{eqnarray*}

In the equation above, $m$ and $n$ denote the start and the end coordinates of a gene in the genome, the top 10% was selected to determine whether the two genes are connected by an edge in the GCN and PNA graph. The gene positions were determined from the GTF v43 release by GENCODE project (23). The tissue-specific 3D genomic distance was determined by mapping gene positions with Hi-C data released by Rao *et al.* (24). Histone modification refers to the chemical alteration of histone proteins, which are involved in packaging DNA into chromatin. It can be considered a type of structural modification that regulates gene expression through a series of biological mechanisms such as acetylation, methylation, phosphorylation, and ubiquitination. We obtained the histone modification data from the NIH Roadmap Epigenomics Mapping Consortium (https://egg2.wustl.edu/roadmap/web_portal/index.html).

### Multi-layer perceptron

A multi-layer perceptron (MLP) model is used as our first deep learning model. The MLP model has three layers, an input layer, a hidden layer, and an output layer. The input layer has a dimension ranging from 800 to 1000 depending on the dataset, the same as the number of selected predictor genes. The hidden layer has a dimension of 2048, the output layer has a dimension ranging from 12 000 to 18 000, the same as the number of mapped genes that is the number of genes in the dataset excluding the predictors genes.

### Auto-encoder

An auto-encoder neural network (25) is used as our second deep learning model. The auto-encoder can map gene expressions to a latent space and reconstruct the gene expressions through fully connected networks. To leverage its ability, a special loss function is used to enforce the reconstruction of predictor gene expressions first and then infer the rest of the genes in the dataset. It was proven later in our tests, that through this special loss function, the auto-encoder has much better performance in mapping genes when missing gene expressions present in the dataset. Our auto-encoder has the following shape: an input layer with dimension as the size as predictor genes, a hidden layer with dimension of 256 (compression), a hidden layer with dimension as the size as the input layer (reconstruction), and an output layer of the size of inferred genes. The special loss function is given as follows:


\begin{eqnarray*}L = \ \frac{1}{n}\mathop \sum \limits_{i = 1}^n {{\left( {{{Y}_i} - {{{\hat{Y}}}_i}} \right)}^2} + \frac{1}{m}\mathop \sum \limits_{j = 1}^m {{\left( {{{X}_j} - {{{\hat{X}}}_j}} \right)}^2}\end{eqnarray*}

The loss function has two terms, the first part being the regular mean squared error loss of the output layer and the second part being the mean squared error between the output of the last hidden layer and the input. The second part of the loss function enforces the reconstruction of the predictor gene expressions (input) before inferring the rest of the genes. The $m$ represents the number of L1000 landmark genes. The $n$ represents the number of predicted genes.

### Graph Convolution Network

Graph Convolution Neural Network (GCN) is a form of Graph Neural Network (GNN). In GCN, each node is processed by a small MLP and then undergoes the message-passing process. This happens multiple times until the desired complexity is achieved. The message passing only happens between nodes that have an edge connecting the two. Different message-passing mechanisms create different GNN models, for example, if the message-passing implements aggregation, then it is considered a GCN. If the message passing implements multiple aggregators with degree-scalers, then it is considered a Principal Neighborhood Aggregation Graph Neural Network (PNA). The node propagation rules and message passing rules for a GCN model are given as follows:


\begin{eqnarray*}X^{\prime} = \ {{\hat{D}}^{ - 1/2}} \cdot \hat{A} \cdot {{\hat{D}}^{ - 1/2}} \cdot X \cdot \theta \end{eqnarray*}

\begin{eqnarray*}X_i^{\prime} = \ {{\theta }^T} \cdot \mathop \sum \limits_{j\epsilon N\left( i \right) \cup \left\{ i \right\}} \frac{{{{e}_{j,i}}}}{{\sqrt {{{{\hat{d}}}_j}{{{\hat{d}}}_i}} }}{{X}_j}\end{eqnarray*}

\begin{eqnarray*}{{\hat{d}}_i} = 1 + \ \mathop \sum \limits_{j \in N\left( i \right)} {{e}_{j,i}}\end{eqnarray*}

In the first equations above, we have the convolution propagation matrix denoted as $X^{\prime}$, $\hat{A}$ is an adjacent matrix, $\hat{D}$ is a diagonal degree matrix, $\theta$ is a weight matrix. $\theta$ can be initialized with different weights to represent edge features. In the second equation above, $X_i^{\prime}$ represents a node in the graph, ${{X}_j}$ represents an adjacent node relative to $X_i^{\prime}$. ${{e}_{j,i}}$ is the edge weight. Through the first equation, the edge weights are convoluted and amplified. Through the second equation information from adjacent nodes can be aggregated to the current node forming message passing. Hence, we call this type of GNN model a Graph Convolution Network (GCN).

### Principal Node Aggregation

PNA is also a form of GNN. The node propagation rules and message passing rules for a PNA model are given as follows:


\begin{eqnarray*}X_i^{\prime} &=& \gamma \left( {{{X}_i},\ {{ \oplus }_{j\epsilon N\left( i \right)}}h\left( {{{X}_i},{{X}_j}} \right)} \right)\ where\ \oplus \\ &=& \ \left[ {\begin{array}{@{}*{1}{c}@{}} 1\\ {S\left( {D,\ \alpha = 1} \right)}\\ {S\left( {D,\ \alpha = \ - 1} \right)\ } \end{array}} \right] \otimes \left[ {\begin{array}{@{}*{1}{c}@{}} \mu \\ \sigma \\ {max}\\ {min} \end{array}} \right]\end{eqnarray*}

In the equation above, ${{X}_i}$ denotes a node, ${{X}_j}$ is the neighboring node. $h,\ \gamma$ denote two MLP models to process the node. ⊕ denotes a combination of scaling and aggregation rules to be applied to the node after the processing of the first MLP $h$ and before the second MLP $\gamma$. The selected scaling and aggregation rules can be determined at the runtime. For maximum compatibility, the scalers selected are ‘linear’, ‘identity’, ‘amplification’, ‘attenuation’. And the aggregators selected are ‘mean’, ‘max’, ‘min’, ‘std’. Through the equation we can see that, the model PNA could have better generalizations across different datasets, simply because the model can learn to use the best combination of scalers and aggregators. Since it is impossible to know which scaler and which aggregator is most suitable for the dataset, the model determines the best combination at the training time.

### Performance evaluation

Models were tested on independent datasets. The standard input is the set of 955 landmark genes from the L1000 method. However, for sensitivity analysis, we removed 70% of gene expression randomly. Three metrics were used to evaluate performance: Pearson's correlation coefficient, mean squared error (MSE) and recall. MSE is transformed using the formula: 2 – exp (MSE). We denote this MSE as tMSE. A higher tMSE denotes better performance. Note that after transformation, tMSE can be negative. Pearson's correlation was calculated between actual gene expression and predicted gene expression for each predicted gene expression. tMSE was computed for all predicted gene expressions. We used the same definition of recall from the L1000 method (26), which was calculated using the following equation:


\begin{eqnarray*}{{R}_i} = \ \frac{{\left| {Z < \ {{\rho }_i}} \right|}}{{\left| Z \right|}}\ with\ Z \sim N(\mu ,\ {{\sigma }^2}\end{eqnarray*}

$Z$ denotes a background set that is also normally distributed. ${{R}_i}$, the recall for gene $i$, is calculated as the percentage of $Z$ smaller than the correlation of gene $i$, denoted as ${{\rho }_i}.$ After we calculated the recall for each gene, we can now calculate the recall for the model using the following equation:


\begin{eqnarray*}{{R}_j} = \ \frac{{\left| {{{R}_i} >\ {{P}_{0.95}}\left( Z \right)} \right|}}{{\left| {{{R}_i}} \right|}}\ for\ \forall i \in \ \left\{ {1,\ \ldots ,n} \right\}\end{eqnarray*}

${{R}_j}$ denotes the recall for model j, and it is calculated as the percentage of recalls for all genes inferred greater than the 95th percentile of the background set. ${{P}_{0.95}}$ denotes the 95th percentile and is equal to 0.66. This testing procedure generated performance measures giving a comprehensive view of the model performances in gene mapping.

### Model training

The model training was conducted on the cluster Pegasus from Frost Institute for Data Science and Computing, University of Miami. Due to the large number of edges involved in the graph features, GPU with large amount of VRAM is required. On a Nvidia A100 GPU with 80GB VRAM, our models on average took ∼50GB VRAM and 12–15 h of training time. Once a model is trained, it takes seconds to run a batch of 32 samples.

## Results

### Overview

The comprehensive study design is guided by two principal objectives. Firstly, we seek to retrace the methodology of the L1000 method, substituting linear regression with deep learning-based approaches. Secondly, we aim to enhance the deep learning models by incorporating graph features designed from regulatory and spatial structural elements. The gene expression prediction models are executed at two distinct levels: normal tissue level and tumor level. For the normal tissue level, training utilized Genotype-Tissue Expression (GTEx) and The Cancer Genome Atlas Program (TCGA) normal tissue data, with independent validation conducted on TCGA normal tissue data. At the tumor level, training employed TCGA breast cancer tumor data, and independent validation was performed on Clinical Proteomic Tumor Analysis Consortium (CPTAC) breast cancer data. Furthermore, Cancer Cell Line Encyclopedia (CCLE) breast cancer cell line data served as a secondary independent validation for tumor expression prediction and generalization to cell lines.

### Generalizability of pair-wise gene correlation

Gene expression prediction operates under the assumption that gene expression patterns exhibit similarity across datasets. However, factors such as tissue specificity and disease conditions profoundly influence gene expression patterns. To systematically account for these factors, we initiated the study with a cross-dataset gene expression similarity analysis. This involved computing pairwise Pearson correlations between genes within each dataset and subsequently evaluating the correlation of correlations for matched gene pairs between distinct datasets. This analysis was carried out pairwise among five datasets: GTEx normal, TCGA normal, TCGA breast cancer tumors, CPTAC breast cancer tumors, and CCLE breast cancer cell lines. Using the same strategy outlined in the L1000 method, for GTEx normal and TCGA normal expression data, we lumped all samples together. To avoid the amount of heterogeneity exhibited in different types of tumors, we selected breast cancer to demonstrate our method.

**Figure 1.**
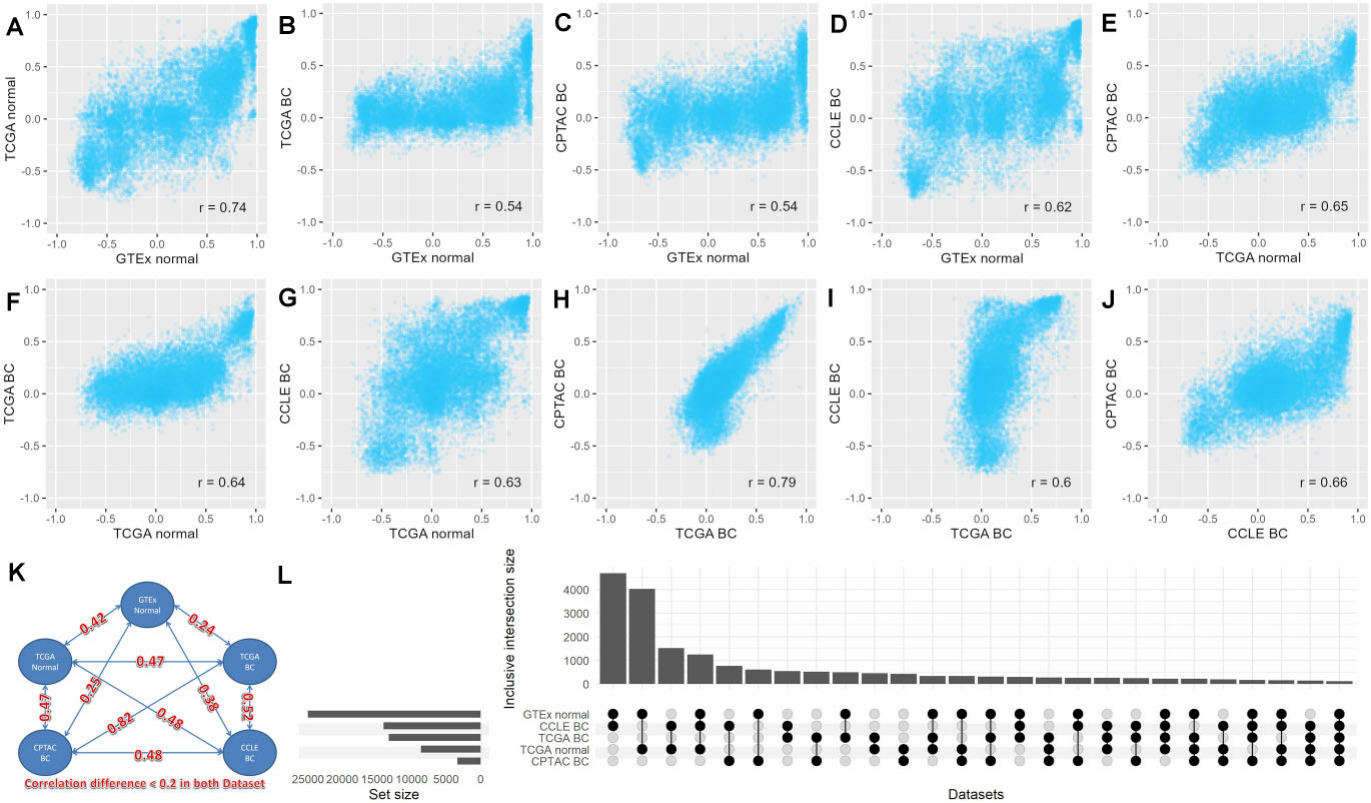
The results from the examination of pair-wise gene correlation generalizability. (**A–J**) Scatter plots of pair-wise gene correlation from one dataset versus the same pair from another dataset. (**K**) A connectivity graph shows the percentage of pair-wise gene correlation that is preserved (Pearson's correlation coefficients > 0.6) between any two datasets. (**L**) An upset plot to show that shared preserved pair-wise gene correlation gene pairs among the tested datasets.

We conducted an assessment to ascertain the generalizability of pair-wise gene correlations by evaluating whether robust correlations could be consistently observed across diverse datasets subjected to variations in tissue types (normal versus tumor). The results revealed the replicability of numerous pair-wise correlations across different datasets; however, it became apparent that the tissue of origin influences the generalizability, as depicted in Figure 1A–J. The findings show that, while many pair-wise correlations exhibit reproducibility across datasets, the tissue-specificity of gene expression introduces a discernible impact on the observed generalizability trends. Notably, the comparison between TCGA breast cancer and CPTAC breast cancer yielded the highest concordance, as evidenced by a Pearson correlation coefficient of 0.79. Similarly, the comparison between GTEx normal and TCGA normal demonstrated the second-highest concordance, with a Pearson correlation coefficient of 0.74. However, it is noteworthy that when matching CPTAC breast cancer tumors with CCLE breast cancer cell lines, the correlation exhibited a notable decrease to 0.66. This reduction in correlation is presumably attributed to the contribution of the microenvironment in bulk tumors. The lowest correlation was observed when aligning tumors with normal tissues, denoted by a Pearson correlation coefficient of 0.54, which is intuitively explicable by the inherent distinctions in tissue types between tumors and normal specimens.

The percentage of gene pairs with correlation differences of <0.2 between any two datasets is depicted on the edges of Figure 1K. The highest percentage (82%) is observed between TCGA BC and CPTAC BC, and the lowest percentage (24%) was observed between GTEx normal and TCGA BC. Using Pearson correlation coefficient ≥ 0.6 as a threshold, the number of shared gene pairs between the datasets is shown in Figure 1L. These findings underscore the consistent maintenance of pair-wise gene expression correlations across diverse datasets. The degree of preservation observed is intricately influenced by the source material, wherein higher correlations are notably more likely to endure among datasets derived from akin sources. These robust correlations, indicative of coordinated gene expression patterns, may be attributed to mechanisms such as co-regulation, the sharing of regulatory elements, physical proximity to the genome, and biological interactions.

### Model comparisons

**Figure 2.**
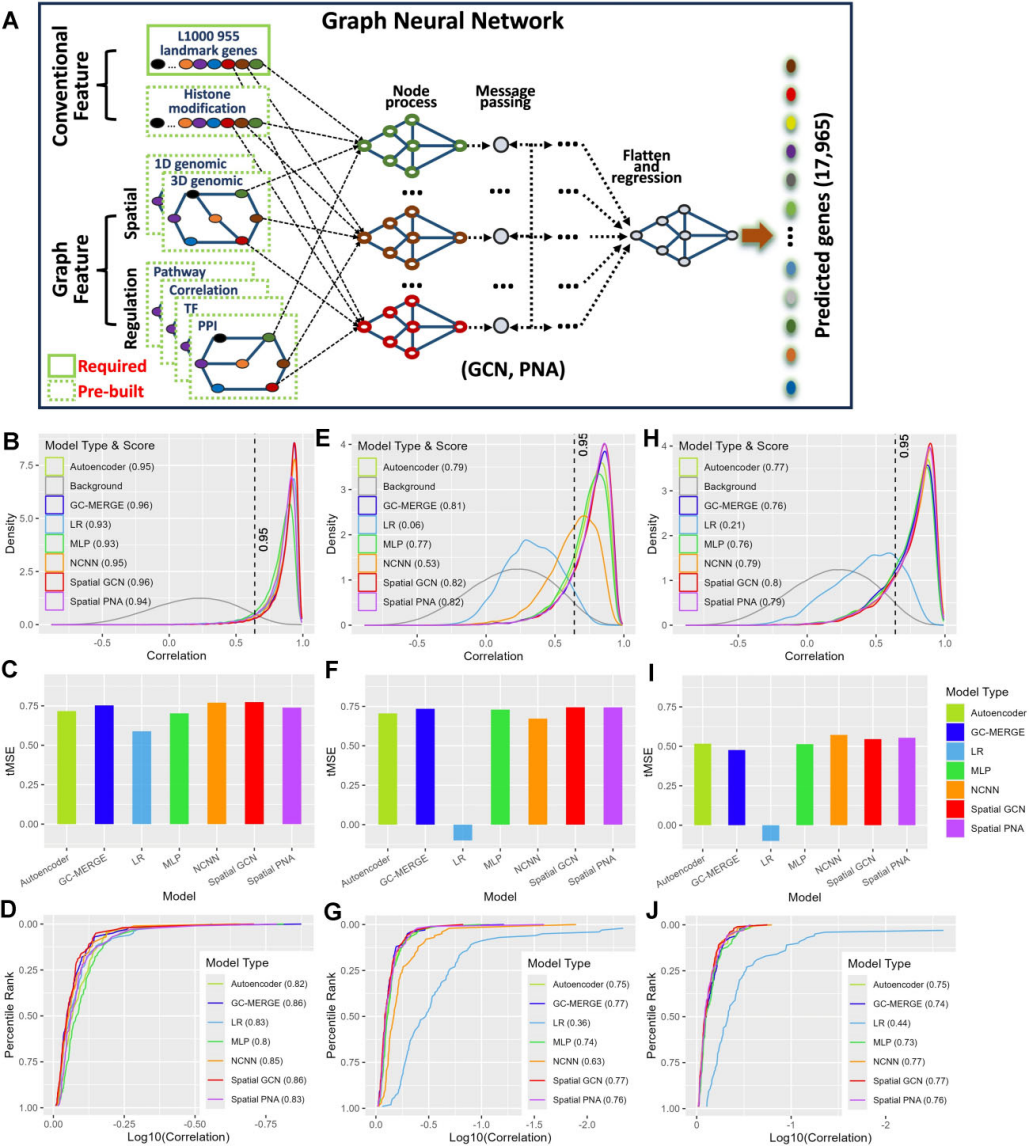
Model design and performance comparison results. (**A**) GNN model design. (**B–D**) Recall, tMSE and correlation results for all models tested using GTEx normal as training and TCGA normal as validation, respectively. (**E–G**) Recall, tMSE and correlation results for all models tested using TCGA BC as training and CPTAC BC as validation, respectively. (**H–J**) Recall, tMSE and correlation results for all models tested using TCGA BC as training and CCLE BC as validation, respectively. For plotting correlation results, the transformation of log_2_(correlation × 1000) was used.

Gene expression similarity is the foundation of the original L1000 method. Our objective was to increase the comprehensiveness of gene connectivity, based on correlation, by incorporating additional features such as regulatory and spatial elements. In the original L1000 method, 12063 samples were obtained from the Gene Expression Omnibus (27) composed of samples from various studies. As the precise composition of the 12063 samples was not specified by the authors, a complete reproduction of this step in the L1000 method is not feasible. Nevertheless, our approach adopts the identical set of 955 landmark genes as determined by the original L1000 method. Moreover, an augmentation of the initially established predictable gene count from 11350 to a maximum of 17 965 has been attempted. In the course of independent validation, 70% of the 955 landmark genes were randomly masked to simulate a real-world scenario reflective of common occurrences of missing data. Figure 2A demonstrates the design of the proposed GNN models. We compared the performance of five models, including two GNNs: Convolution Network (GCN) and Principe Node Aggregation (PNA); two non-graph neural network models: Multi-layer Perceptron (MLP) and Auto Encoder (AE); and One basic statistical approach: Linear Regression (LR). In addition to these five models, we also compared our method to NCNN[10] and GC-MERGE (11).

To make the comparison fair, we tried to use similar settings as the models permit. The models were fitted with the appropriate features in the following manner. All models were fitted with gene expressions in the original L1000 method's framework. The two graph neural network models, GCN and PNA were fitted with gene expression, pair-wise correlation, and chromosomal genomic distance (Materials and methods). NCNN was fitted with PPI as dictated by the original method. GC-MERGE was fitted with histone modification and Hi-C as required original method. The performance of these models was assessed using recall, transformed mean squared error (tMSE), and Pearson correlation metrics (Methods). In the scenario of mixed normal tissue, where GTEx normal tissue expression data as the training set and TCGA normal tissue data as the validation set, all models demonstrated comparable recalls, as illustrated in Figure 2B. GC-MERGE and GCN achieved the highest recall, both registering at 0.96, while LR and MLP attained the lowest recall at 0.93. For tMSE evaluation (Figure 2C), GCN obtained the highest tMSE at 0.77, whereas LR recorded the lowest tMSE at 0.59. In terms of overall correlation (Figure 2D), GCN and GC-MERGE secured the highest correlation coefficient of 0.86, whereas MLP garnered the lowest correlation at 0.8.

In the scenario of cancer tissue, where TCGA Breast Cancer (BC) was utilized as the training set and CPTAC BC served as the validation set, it is noteworthy that GCN and PNA achieved the highest recall values, both attaining 0.82, while LR exhibited the lowest recall at 0.06 (Figure 2E). The models' performance was further delineated by tMSE metrics, with GCN and PNA both registering the highest tMSE at 0.74 and LR obtaining the lowest tMSE (<0) (Figure 2F). NCNN and GC-MERGE performed slightly worse registering 0.67 and 0.73 respectively. In terms of overall correlation, GCN and GC-MERGE demonstrated the highest correlation coefficient of 0.77, whereas LR exhibited the lowest correlation at 0.36 (Figure 2G). In a separate assessment utilizing TCGA BC as the training set and CCLE BC as the validation set, the models again showcased distinct performance characteristics. GCN achieved the highest recalls at 0.8 with LR recording the lowest recall at 0.21 (Figure 2H). Both PNA and NCNN performed well registering 0.79. Examining tMSEs revealed that NCNN achieved the highest tMSE at 0.57, while GCN and PNA achieved the second-best values at 0.55. LR showed the worst performance. (Figure 2I). Notably, in terms of overall correlation, NCNN and GCN garnered the highest correlation coefficient of 0.77, while LR displayed the lowest correlation coefficient of 0.44 (Figure 2J).

Additionally, we conducted a comparative analysis between our approach and previous methods (NCNN, GC-MERGE, and Enformer) across various aspects including study design, feature selection, input requirements, and output types (Table 1). In terms of study design, Enformer employs a transformer architecture, while our method, NCNN and GC-MERGE are all based on graph neural networks. Notably, our study and Enformer considered tissue specificity and employed independent validation, whereas NCNN and GC-MERGE do not. Additionally, we took into account the model's portability across tumor samples, cell lines, and normal tissues. Regarding non-graph features, Enformer solely utilizes DNA sequences, limiting its applicability in real-world scenarios. Conversely, GC-MERGE and our method also incorporate histone modification data. In terms of graph features, NCNN utilizes protein–protein interaction (PPI) data, GC-MERGE employs Hi-C data, and our method incorporates PPI, transcription factors (TF), Hi-C, 1D genomic distance, pathway information, and pair-wise correlation. In terms of required input, Enformer solely requires DNA sequences, which serves as both an advantage and disadvantage. Our method, NCNN and GC-MERGE require partial gene expression data. Regarding output, Enformer predicts a 200 kbp track and then converts it to gene expression, while GC-MERGE predicts a 10 kb track before conversion. In contrast, our method and NCNN directly predict gene expression levels.

**Table 1:** Comparison to previous gene expression prediction methods

Study PubMed ID	34608324 (Enformer)	36745614 (NCNN)	35325548 (GC-MERGE)	NA (Ours)
Study Design				
Neural network model	Transformer	Modified MLP	GCN	GCN/PNA
Graph-based	No	Yes	Yes	Yes
Independent validation	Yes	No	No	Yes
Cancer tissue analysis	No	No	No	Yes
Tissue-specific analysis	Yes	No	No	Yes
**Non-graph features**				
Personalized DNA sequences	✓			
Gene expression		✓	✓	✓
Histone modification			✓	✓
**Graph features**				
PPI		✓		✓
Transcription factor				✓
3D Hi-C			✓	✓
1D genomic distance				✓
Pathway				✓
Pair-wise correlation				✓
**Required input**				
DNA sequence	✓			
Partial gene expressions		✓	✓	✓
**Output**				
Prediction unit	200 KB	Gene	10 KB	Gene

These findings serve to illustrate that gene expression exhibits greater stability and can be easily modeled in the context of normal tissue. Although models trained using tumor tissues and cell lines can perform well, their proficiency tends to be comparatively diminished when contrasted with models trained on normal tissue. This disparity in performance is attributed to the greater variability introduced by the unique characteristics of tumors. It is noteworthy that GNNs, in particular, demonstrated a distinctive advantage due to the integration of additional features that are not attainable through LR and non-GNNs. This inherent capability allowed GNNs to achieve superior performance, underscoring the significance of their ability to leverage graph structures for more comprehensive modeling in the domain of gene expression prediction.

### Graph features

**Figure 3.**
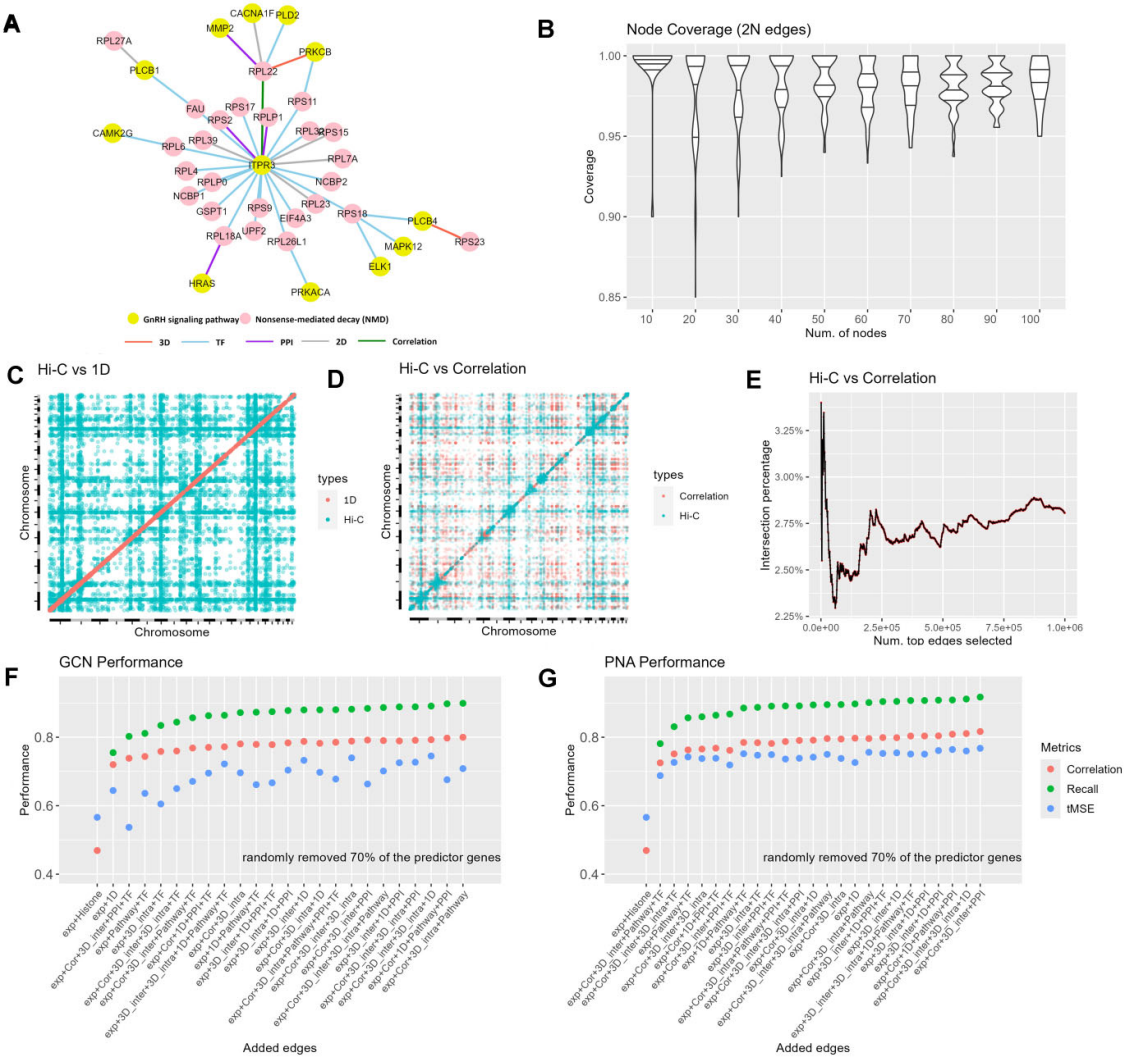
Performance evaluation results for GCN and PNA when adding additional graph features. (**A**) example of how genes are interconnected between the GnRH signaling and nonsense-mediated decay pathways. Only partial genes were drawn for demonstration. (**B**) After simulating 1000 iterations from 10 to 100 nodes, using 2N edges consistently increases the likelihood of a path connecting all nodes in the graph. (**C**) A heatmap that shows genome-wide overlapping hot spots between genomic Hi-C 3D distance and chromosomal distance. (**D**) A heatmap that shows genome-wide overlapping hot spots between 1D genomic and Hi-C 3D distance. (**E**) A plot that shows the overlapping proportions of gene pairs between Hi-C genomic distance and pair-wise gene correlation. This plot shows that Hi-C 3D distance and pair-wise gene correlation capture distinct genomic information. (**F**) GCN performance measured using GTEx normal as training and TCGA normal as validation with different combinations of features. (**G**) PNA performance measured using GTEx normal as training and TCGA normal as validation with different combinations of features.

Building upon the above results, we have substantiated the higher efficacy of GNNs. This superiority is predominantly ascribed to their inherent capacity to integrate multiple features. As a further improvement, here we focus on incorporating additional graph features encompassing biological pathways, PPI networks, (transcription factor) TF regulation and 3D genomic distances. The augmentation of these features contributes to a more comprehensive detection of the gene-to-gene relationships within the graph. For instance, we consider the GnRH signaling pathway (KEGG.HSA.04912) and nonsense-mediated decay (REACT.R.HSA.975957) as examples. The genes within each pathway are interconnected through regulatory effects, while the genes spanning the two pathways are linked via pair-wise gene correlation, chromosomal and 3D genomic distances (Figure 3A). This strategic enhancement facilitates a richer representation of the intricate relationships among genes within the broader biological context.

The typical pathway encompasses a range of genes varying from 10 to 100. Due to the unavailability of precise connection edges and recognizing that a fully connected graph would necessitate (*N*(*N*– 1))/2 edges, where *N* represents the number of genes, opting for such an approach imposes substantial load on GPU memory resources. In light of this, we have chosen to employ a heuristic solution involving the random selection of 2*N* edges to establish connections among genes within a given pathway. Through simulation of 1000 iterations, wherein the node count starts at 10 and incrementally progresses to 100 nodes, the results demonstrate that utilizing 2*N* edges yields a favorable probability of ensuring the existence of a path connecting all nodes within the graph (Figure 3B).

Our preliminary investigation is oriented towards the systematic evaluation of the applicability of graph features. Notably, chromosomal distance is computed within the limits of intra-chromosomal interactions, whereas 3D distance encompasses both intra and inter-chromosomal interactions. In the context of our analysis, the comparison involves top Hi-C 3D distances (Top 100 000 for intra and 100 000 for inter-chromosomal), providing insights that are not encompassed by chromosomal distance (top 10%) (Figure 3C). The selection of the Top 100 000 Hi-C records for intra-chromosomal interactions is dictated by GPU memory limitations. Pair-wise gene correlations were calculated without the imposition of chromosome restrictions, which potentially has extensive overlapping with Hi-C 3D distance. However, our findings suggest that despite the presence of shared intra-chromosomal genomic hotspots between correlation and Hi-C data, a considerable amount of distinct information is captured by each metric (Figure 3D). Indeed, our results demonstrate that the highest overlapping proportion between the gene pairs identified by top Hi-C 3D distance and pair-wise gene correlation is 3.25%. This substantiates the assertion that Hi-C 3D distance encapsulates additional information compared to pair-wise gene correlation, as depicted in Figure 3E. For example, for the graph with 30 nodes, the median probability of a pathway that covers all 30 nodes is 98% with 2*N* random edges.

Subsequently, we conducted an assessment involving various feature combinations within the GCN and models. The foundational feature in this analysis is gene expression, supplemented by additional features encompassing pair-wise gene correlation, biological pathway information, chromosomal genomic distance, and 3D genomic distance. The 3D genomic distance is further divided into intra- and inter-chromosomal. Furthermore, the incorporation of biological pathway, PPI network, TF regulation and Hi-C 3D genomic distance as additional features demonstrated a notable enhancement in the performance of both models. For example, in GCN models, the edge combination of ‘expression + correlation’ achieved 0.8, 0.74 and 0.7 for recall, correlation, tMSE, respectively. After adding inter-chromosome 3D genomic distance edges, the recall, correlation, and tMSE increased to 0.9, 0.8 and 0.75, respectively. in PNA models, the edge combination of ‘expression + intra-chromosome 3D genomic distance edges + 1D genomic distance edges’ achieved 0.89, 0.79 and 0.74 for recall, correlation, tMSE, respectively. After adding biological pathways, the recall, correlation, and tMSE increased to 0.91, 0.81 and 0.78, respectively. Our results also revealed superior overall performance of the PNA model (Figure 3F) in comparison to the GCN model (Figure 3G). On average, PNA models performed 2%, 1% and 9% better than GCN for recall, correlation and tMSE, respectively. The complete results including more combinations of features not included in Figure 3F and G are available in Supplementary Table S1.

**Figure 4.**
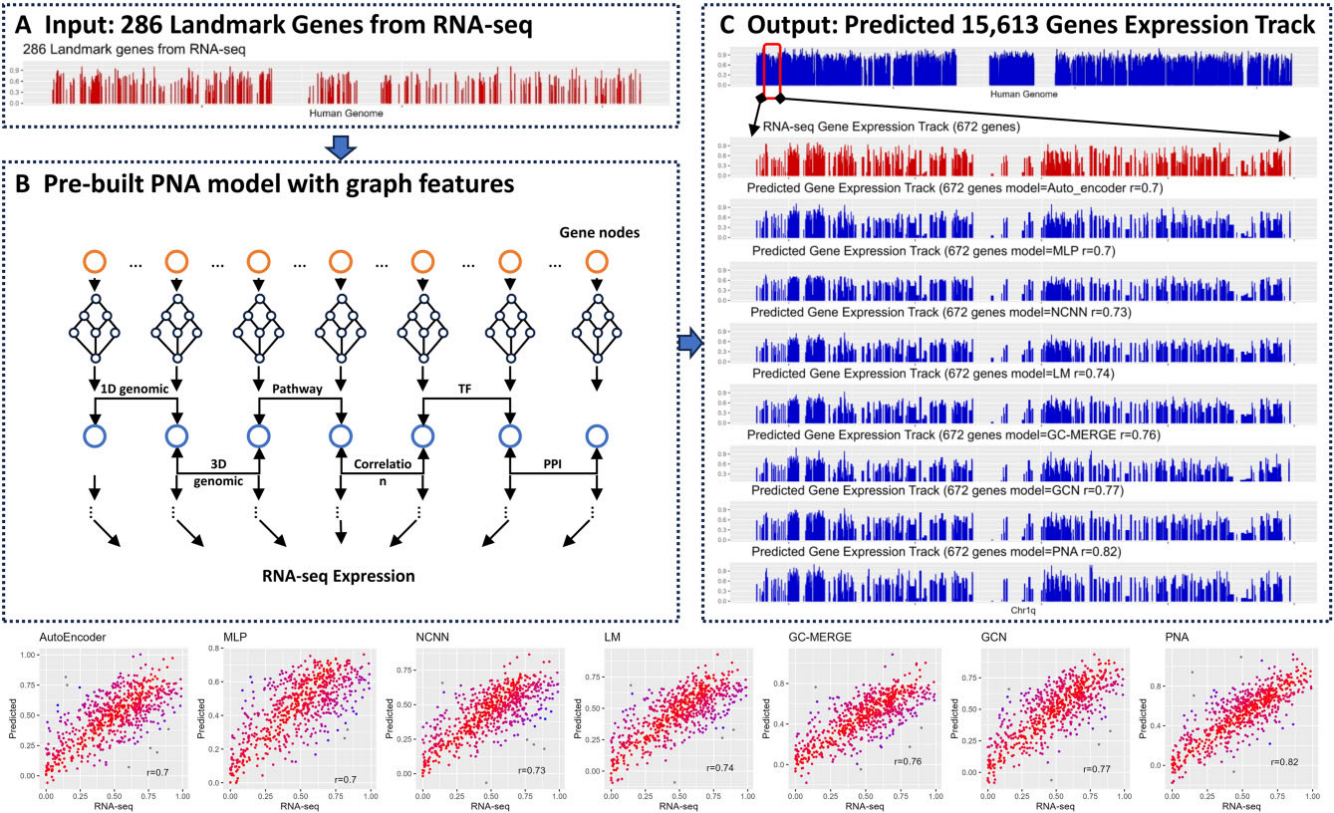
An example of an application using our PNA model on one subject (TCGA-BH-A0DP). (**A**) The RNA-seq gene expression track for the 286 genes randomly selected from the 955 original landmark genes. (**B**) The PNA model was trained using GTEx normal gene expression with graph features. (**C**) The gene expression of 15 613 genes were predicted. Zooming into chr1q, 672 genes were predicted, and their track is compared to gene expression track from RNA-seq. Only predicted genes are plotted on the gene expression tracks.

To illustrate the efficacy of GNN, we present a specific example involving the utilization of the PNA model trained with GTEx normal gene expression data. As a case study, we employed the model to predict gene expression for the subject TCGA-BH-A0DP. Subsequently, 70% of the 955 landmark genes were masked randomly, leaving 286 genes as input (Figure 4A). Following the application of the PNA model (Figure 4B), a total of 15613 genes were successfully predicted. The performance metrics for this instance demonstrated a recall of 0.92, a correlation coefficient of 0.81, and a tMSE of 0.76. For a more granular examination, we directed our attention to chromosome 1q, where 672 genes were predicted. Notably, the predicted tracks and RNA-seq track for these 672 genes exhibited similar patterns for all models tested, minor details can be observed when examining closely (Figure 4C). The major performance on prediction of these 672 genes among the different models can be better depicted through correlation in scatter plots (Figure 4D). The PNA model achieved the highest Pearson correlation coefficient at 0.82. Collectively, these findings underscore that, in the majority of the test scenarios, the incorporation of additional graph features led to an improvement in performance. This emphasizes a significant advantage of GNN over traditional neural networks.

### Tissue specificity

**Figure 5.**
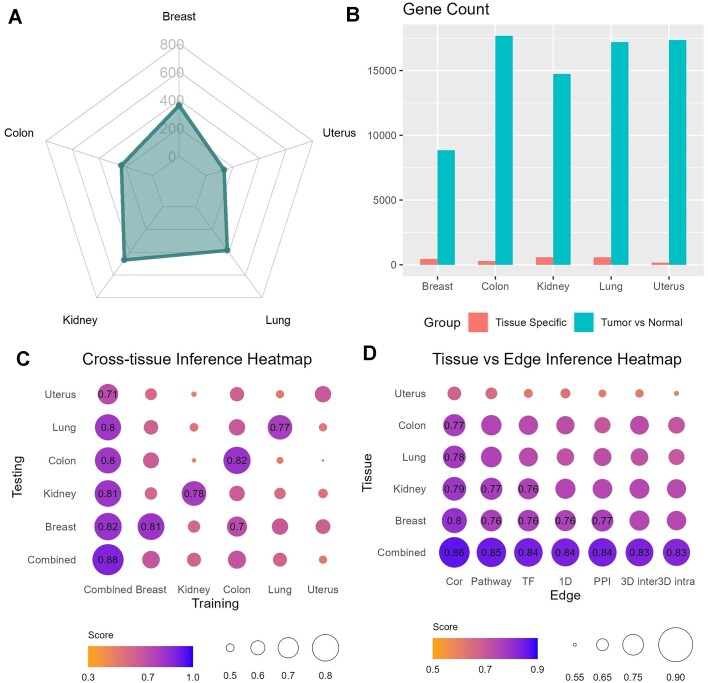
Tissue specificity analysis. (**A**) The number of tissue specific genes are shown in a radar plot. (**B**) In comparison, the number of differentially expressed (≥3× fold change) genes between tumor and normal of the same tissue type is much greater than the number of tissue-specific genes. (**C**) The cross-tissue performance scores (average of recall, tMSE and correlation) are represented in a heatmap matrix format. Combined, tissue-agnostic model performed the best overall. (**D**) Performance of single graph feature in tissue specific setting shows that best performance was achieved in the tissue-agnostic model.

Gene expressions are recognized to exhibit tissue-specific patterns, although the proportion of truly tissue-specific genes is limited (28). We investigated whether tissue-specific genes could introduce any performance challenges in gene expression prediction across five tissue types: breast, colon, liver, lung, and uterus. Tissue-specific genes were defined as those with expression levels at least three times higher than those in any other tissue. We utilized GTEx data to discern tissue-specific gene expressions and TCGA’s normal data for validation. Across the five chosen tissue types, we identified a total of 1970 tissue-specific genes (breast: 451; colon: 291; kidney: 584; lung: 476; uterus: 166) (Figure 5A). On average, there are 394 tissue-specific genes per tissue type, which constitutes a small proportion of the entire transcriptome. Among these, 6 genes overlap with L1000’s landmark genes. Notably, the number of differentially expressed genes between tumor and normal tissue of the same type far exceeds the count of tissue-specific genes (Figure 5B), suggesting that disease-related variation surpasses that of tissue specificity.

We conducted independent cross-tissue model validation using PNA model, wherein tissue-agnostic and tissue-specific models were evaluated against both intended and non-intended tissue types. The evaluation metrics (recall, correlation and tMSE) were averaged to represent the overall performance (Figure 5C). Overall, the tissue-agnostic model outperformed in all scenarios except for colon, where the colon-specific model exhibited slightly better performance in predicting colon tissue gene expression. Additionally, we assessed the contribution of graph features by tissue type using the PNA model and found that pair-wise gene correlation had the most significant impact, followed by pathway, across all tissue types (Figure 5D). All features had greater contributions in the tissue-agnostic setting than in tissue-specific settings. This result also implies that predicting gene expressions in certain tissues poses greater difficulty compared to others, possibly attributable to the tissue-specific characteristics of certain features. For instance, it is established that a fraction of transcription factor regulation exhibits tissue specificity [ (29]. Nevertheless, the presence of a non-tissue-specific portion of the features is adequate to facilitate the commendable performance of the PNA model. Furthermore, the variation caused by carcinogenesis surpasses the variation observed between different normal tissue types. While the limited number of tissue-specific genes serves certain purposes such as cell-free RNA tissue type deconvolution (30), they do not introduce sufficient variation to affect our mixed tissue gene expression prediction model.

## Discussion

Genes in the human genome are connected as a complex and intrinsic web. The relationship among genes has been a valuable analytical tool in genomics. It can be used for co-expression analysis, biological pathway/network analysis, functional annotation, biomarker discovery, sample quality control, drug response prediction, etc. Gene expression prediction is an established field, and the foundation is described by the L1000 method (8), which was primarily based on the multi-variable linear regression model. Furthermore, it was demonstrated that the prediction of gene expression can be achieved by integrating long-range interactions using the Enformer tool (9). Notably, this tool enables the prediction of gene expression solely from genomic sequences, eliminating the requirement for a partial transcriptome, a distinction from methodologies such as L1000 and our proposed method. The merit of this approach lies in its independence from a partial transcriptome dataset. However, a limitation of this approach is that the accurate capture of genuine gene expression variation necessitates the availability of personalized genomes with individualized DNA sequences. The practical challenge arises from the often intricate and less readily accessible nature of de novo assembled sequences compared to the more direct acquisition of gene expression data from RNA-seq experiments. In contrast, our approach capitalizes on the incorporation of additional graph features, a strategic augmentation that contributes to heightened predictive performance. Importantly, these graph features are derived from previously generated independent datasets, mitigating the requirement for personalized DNA sequences. Consequently, our method necessitates solely a partial transcriptome as input, in our case, up to 70% fewer landmark genes from the L1000 method, circumventing the challenges associated with obtaining individualized genomic sequences encountered in alternative methodologies.

In the scope of this investigation, our primary objective is to enhance the modeling of gene-to-gene relationships through the utilization of GNNs. This strategic choice stems from the demonstrated superior performance of such networks compared to traditional approaches, attributed to their adept incorporation of graph features. Given our emphasis on gene expression as the primary outcome, the inclusion of genomic DNA sequences as features becomes inherently incongruous. This arises from the homogeneity in genomic sequences across all subjects when starting from a reference genome. Consequently, the use of genomic DNA sequences as features in the context of this study lacks substantive relevance. On the other hand, the inclusion of genomic sequences as features would potentially yield greater utility in a Pan-Genome setting where individual genomic sequence variations are meticulously documented. However, the sample size limit of current available pan-genomes prohibits the generation of a proper model.

The focal point of our investigation centered on two GNNs: Graph GCN and PNA. In comparison to LR, Autoencoder, and MLP, GCN and PNA exhibited superior performance during the initial stages of evaluation. This enhanced performance can be attributed to the unique capability of GCN and PNA to assimilate chromosomal genomic distance and pair-wise gene correlation as graph features, a capacity not shared by LR, Autoencoder, and MLP. Furthermore, the advantage of GCN and PNA was notably accentuated by the incorporation of additional graph features including biological pathway information, PPI networks, TF regulation, and Hi-C inferred 3D genomic distance. The cumulative effect of these augmented graph features contributed to the heightened performance observed in GCN and PNA.

Compared to previous methods of gene expression prediction, Enformer (9), NCNN (10), GC-MERGE (11). our study has the following advantages: (i) under the same settings, our method performed similarly or better; (ii) our method achieved better performance by accounting for more biological connectivity through additional graph features; (iii) the issue of tissue specificity was accounted for, thus our model performs more consistently across multiple tissue or data types; (iv) disease-specificity was analyzed regarding cancer; (v) independent validation was conducted instead of cross-validation. (vi) Unlike NCNN where edges were hardcoded into the model, GCN and PNA’s edges input can be flexible.

One inference drawn from our analyses is the notable significance of gene expression variation induced by carcinogenesis, which can exceed the variance observed among distinct normal tissue types, aligning with prior findings (28,31). Additionally, our investigation revealed that the proportion of tissue-specific genes is relatively small within the entire transcriptome. Consequently, the advantage derived from aggregating all tissue types with a larger sample size outweighs the variation introduced by tissue-specificity, a rationale likely akin to the decision made by the original L1000 method (8) to amalgamate all tissue types.

The GNNs have several advantages over traditional neural networks. GNNs are better suited for capturing intricate relationships and dependencies among data points. This is especially effective when the relationship between entities matters, such as pair-wise gene correlation and regulation from a biological pathway between two genes. No graph neural network can work with graph features by either modifying the neural network or the graph feature. However, this is an inefficient approach and produces inferior results as we have shown in NCNN. These complicated relationships are easier to incorporate into GNNs by representing them as a graph rather than text or numerical matrix. In our study, individual genes are treated as nodes, with their respective expressions serving as the features of the nodes. The relationships between genes are represented as edges, forming a comprehensive graph that encapsulates the entire gene expression dataset. The distinctive feature of GNNs lies in their inherent capacity for message passing between nodes, enabling the seamless transfer of information among related genes irrespective of distances or boundaries. The diverse information aggregation techniques employed in GNNs play a pivotal role in filtering and enhancing the information arriving at each node. This attribute renders GNNs particularly adept at robustly mapping gene expressions. Additionally, GNNs exhibit resilience to noisy or incomplete data, attributable to their unique capabilities in message-passing aggregation. The inherent robustness and information processing capabilities of GNNs contribute to their efficacy in the nuanced mapping of gene expressions within complex datasets. Traditional neural networks may be more sensitive to data irregularities, especially when dealing with structured data like graphs. Compared to the L1000 method, we demonstrated the superior performance of GNNs. GCN and PNA performed notably better than LR primarily due to the ability to incorporate additional graph features that can better describe the gene-to-gene relationship. Moreover, the increased performance can also be partially due to the linearity agnostic approach which we discussed earlier. Furthermore, since gene expressions are tissue specific (32), the real-world application model could benefit more from tissue-specific training. Also, in order to compare to L1000 method, we tried to reproduce the setting of L1000 method by using the original 955 landmark genes. Ideally, selecting novel landmark genes can potentially further increase the number of predictable genes and performance.

In our analyses, we focused on GCN and PNA. GCNs typically perform a localized convolutional operation on each node, aggregating information from neighboring nodes. The convolutional operation is often based on a normalized version of the graph Laplacian matrix. GCNs aggregate information by taking a weighted sum of the features of neighboring nodes. The weights are determined by the edges in the graph. PNA, on the other hand, uses an aggregation scheme that combines the information from neighboring nodes by considering both the individual nodes and their pairwise interactions. It includes self and neighbor-aggregation terms. PNA uses learnable aggregation weights that capture the importance of different nodes and interactions. This allows the model to adaptively determine the significance of various elements in the aggregation process. Furthermore, PNA explicitly models higher-order interactions by considering both the node and pairwise features. This enables the model to capture more complex relationships in the graph. In summary, while both GCN and PNA are GNNs, they differ in their aggregation schemes and mechanisms. GCN relies on a convolutional operation and aggregation based on direct neighbors, while PNA introduces a more expressive aggregation scheme considering both node and pairwise interactions with learnable weights. In our assessment, PNA demonstrated overall better performance than GCN in the setting of gene expression prediction. While GNNs offer these advantages, it's essential to note that their effectiveness depends on the nature of the data and the specific task at hand. In scenarios where the data can be naturally represented as a graph, GNNs often outperform traditional neural networks. However, for tasks with sequential or grid-like data, traditional neural network architectures may still be more suitable.

## Supplementary Material

gkae514_Supplemental_Files

## Data Availability

All codes, models and related material can be found at https://github.com/yay135/gene_connectivity and https://figshare.com/projects/Project_Gene_Connectivity/206998 (code: https://doi.org/10.6084/m9.figshare.25934029.v2, data: https://doi.org/10.6084/m9.figshare.25934185.v1). A Docker for our model has been prepared and can be downloaded at the same GitHub link.
